# Feasibility and acceptability of home-based management of malaria strategy adapted to Sudan's conditions using artemisinin-based combination therapy and rapid diagnostic test

**DOI:** 10.1186/1475-2875-8-39

**Published:** 2009-03-09

**Authors:** Khalid A Elmardi, Elfatih M Malik, Tarig Abdelgadir, Salah H Ali, Abdalla H Elsyed, Mahmoud A Mudather, Asma H Elhassan, Ishag Adam

**Affiliations:** 1 National Malaria Control Programme, Federal Ministry of Health, Khartoum, Sudan; 2 Communicable Disease Control Administration, Federal Ministry of Health, Khartoum, Sudan; 3 Khartoum Ministry of Health, Khartoum, Sudan; 4 University of Khartoum, Faculty of Medicine, Khartoum, Sudan

## Abstract

**Background:**

Malaria remains a major public health problem especially in sub-Saharan Africa. Despite the efforts exerted to provide effective anti-malarial drugs, still some communities suffer from getting access to these services due to many barriers. This research aimed to assess the feasibility and acceptability of home-based management of malaria (HMM) strategy using artemisinin-based combination therapy (ACT) for treatment and rapid diagnostic test (RDT) for diagnosis.

**Methods:**

This is a study conducted in 20 villages in Um Adara area, South Kordofan state, Sudan. Two-thirds (66%) of the study community were seeking treatment from heath facilities, which were more than 5 km far from their villages with marked inaccessibility during rainy season. Volunteers (one per village) were trained on using RDTs for diagnosis and artesunate plus sulphadoxine-pyrimethamine for treating malaria patients, as well as referral of severe and non-malaria cases. A system for supply and monitoring was established based on the rural health centre, which acted as a link between the volunteers and the health system. Advocacy for the policy was done through different tools. Volunteers worked on non-monetary incentives but only a consultation fee of One Sudanese Pound (equivalent to US$0.5).

Pre- and post-intervention assessment was done using household survey, focus group discussion with the community leaders, structured interview with the volunteers, and records and reports analysis.

**Results and discussion:**

The overall adherence of volunteers to the project protocol in treating and referring cases was accepted that was only one of the 20 volunteers did not comply with the study guidelines. Although the use of RDTs seemed to have improved the level of accuracy and trust in the diagnosis, 30% of volunteers did not rely on the negative RDT results when treating fever cases. Almost all (94.7%) the volunteers felt that they were satisfied with the spiritual outcome of their new tasks. As well, volunteers have initiated advocacy campaigns supported by their village health committees which were found to have a positive role to play in the project that proved their acceptability of the HMM design. The planned system for supply was found to be effective. The project was found to improve the accessibility to ACTs from 25% to 64.7% and the treatment seeking behaviour from 83.3% to 100% before- and after the HMM implementation respectivly.

**Conclusion:**

The evaluation of the project identified the feasibility of the planned model in Sudan's condition. Moreover, the communities as well as the volunteers found to be satisfied with and supportive to the system and the outcome. The problem of treating other febrile cases when diagnosis is not malaria and other non-fever cases needs to be addressed as well.

## Background

Despite the great efforts exerted by the national and international communities, malaria is still one of the major public health problems [1]. Recently the World Health Organization (WHO) has advocated for expansion and acceleration of malaria elimination efforts [2-4]. To achieve this, the problem of accessibility to effective treatment should be solved, more emphasis should be directed towards confirmed malaria diagnosis, and coverage with suitable protective measures should be greatly increased.

Effective management of malaria requires that the consumers and the care-givers, seek, obtain, and use drugs appropriately [5]. This should be linked to timely decision, accessibility, correct use of the drugs and follow-up after prescription. Malaria in children under five years of age requires mothers to recognize early the malaria symptoms, in particularly fever. This recognition in addition to classification by caregivers is a key to intervention [6]. In this context, Home Malaria Management (HMM) has become one of the important strategies to reach this precious target of malaria elimination [7,8].

Inaccuracy in confirmation of malaria status using microscopy can lead to patient mismanagement. Although diagnosing malaria solely on the clinical feature can reduce the risk of missing malaria cases, but it can lead to over-treatment and thus increasing the pressure on drugs and predisposing to appearance of resistant strains. Using Rapid Diagnostic Tests (RDT) may show evidence of cost-effectiveness for this approach [9,10].

Malaria in Sudan was determined as one of the most devastating problem that led to loss of 2,877,000 DALYs in the year 2002 and was identified as a common cause of fever [11,12]. Possible contributing factors leading to this grave situation include floods, draught, famine, widely extended irrigated schemes without enough consideration to health component and population movement (internal displacement and influx of refugees). The situation may be further aggravated by insecticide resistance and by the spread of *Plasmodium falciparum *resistant strains [13-15]. On a background of a widespread chloroquine resistant *P. falciparum*, Sudan has changed its treatment policy to artemisinin-based combination therapy (ACT) in 2004, with artesunate plus sulphadoxine-pyrimethamine (AS/SP) as the first-line treatment for uncomplicated malaria. This did not solve the problem since accessibility to health services is still a barrier.

Late consultation (in average 3 days) for fever/malaria treatment is another barrier [16]. Mothers with febrile children seek care at home with what available at hand (herbs, remaining drugs, drugs from shops, and tepid sponging) and then shifted to health workers if there is no response or if the condition worsened. This in addition to low coverage with health facilities, dissatisfaction with services, difficulty to reach the facilities especially during rainy season, believes and satisfaction with traditional medicine and herbs, and user fees represents important contributing factors to late consultation. Another two things influencing consultation were the severely ill child need urgent consultation and hence shorten the duration; and appearance of illness at night deter the child from health facilities care waiting for the sun to rise and hence prolong the duration. This practice was similar to what reported by others [6,17-19]. Self-treatment was common as stated by community leaders and health personnel in the area, which didn't vary from results obtained from central Sudan [20].

The aim of this research was to evaluate the feasibility and acceptability of HMM via volunteers using ACT and RDTs in Sudan's condition. Special focus of this study was laid on assessing the followings: volunteers performance in terms of compliance with the study treatment guidelines, RDT use, referral instructions, registration and reporting; volunteers satisfaction with the proposed motivation scheme; feasibility of the designed system for medicines and commodities supply, distribution and storage; and acceptability of the planned HMM system by the community.

## Methods

### Study settings

**Figure 1:**
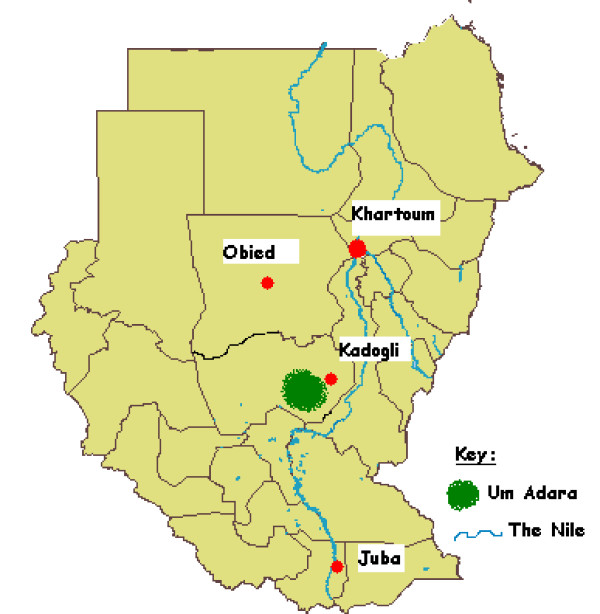
**Map showing Sudan and Um Adarah area**.

During the period May 2007 and January 2008, the study was conducted in Um Adara area in South Kordofan state (Figure 1). The area is composed of 43 villages with an average population of around 75,000 including nomadic. Nomadic has a seasonal north and south movement tracing water sources (rain fall) and grass for their cattle. The area is classified as hypo- to meso-endemic -as per the National Malaria Control Programme stratification. Malaria transmission is associated mainly with the rainy season, which extends on average from May to November. In general the access to villages in the area is somewhat difficult notably in the rainy season. Twenty villages in this area were selected because of the availability of previous information regarding treatment seeking behaviour [16] and relative accessibility for supervision purposes.

### Subject and intervention

The study was designed to provide malaria diagnosis and treatment services to the communities as close to their residence as possible through a volunteer provider named as "Malaria Control Assistant" [MCA]. MCAs were selected -one per village- by their communities according to specified characteristics provided by the study team including availability in the area most of the time, acceptability by the community, reading and writing ability, and willingness to work as volunteer. Training of MCAs was focused on identification of malaria cases using rapid diagnostic test (RDT) and treating them with AS/SP, identification of severe malaria clinical presentation and giving pre-referral treatment in form of artesunate suppositories, and picking up other danger signs and non malaria cases for referral. Recording and reporting of information related to the cases was also addressed, as well as stock management. Training was done for seven days by medical doctor and laboratory technician working in the Malaria Control Programme. A simple training module was developed by the study team that involved problem solving, group work, exercises, demonstration and practical sessions.

Drugs and RDTs used for the project were assessed and found to be satisfactory in their stability and performance, respectively, as their assessment results revealed. ACTs stability was done in the National Drugs Quality Control Laboratory. The overall efficacy of AS/SP in Sudan was 98.5% in 2007 as per the National Malaria Control Programme anti-malarial drugs monitoring results. AS/SP used for the study were from Guilin Pharmaceutical Co., China; and the artesunate suppositories were from Mepha, Switzerland. RDTs readings were blindly checked against microscopy examination for the same patients by different laboratory technician in the Central Malaria Reference Laboratory, which showed a 97% and 100% sensitivity and specificity respectively. RDTs were brought for the study from Acon Laboratories, United States of America. The malaria parasitological prevalence was done in the same communities and individuals targeted by the HHS, using microscopy examination. Blind cross-reading of slides took place for quality assurance purposes.

MCAs worked on non-monetary incentives but only a One Sudanese Pound (equivalent to US$0.5) consultation fee, which was assured to be reasonable when discussed with the community leaders. So no salaries were arranged for MCAs and they work as volunteers on this base. No other mean of incentive was used rather than the community respect.

To assure adequate understanding and adherence to the project guidelines, monthly supervisory visits and meetings -including clinical cases and problems discussions- in the first three months took place; then weaned over time. Supplies and essential equipments were provided free through the nearest health facility, which became the link between MCAs and the ordinary health system.

Sensitization and advocacy for the communities to use the HMM services was started earlier in the process in each village. Community leaders played a great roll in this process. The graduation of MCAs was attended by a well community representation, when it became a chance to further advocate for the project. Thereafter, an advocacy campaigns were done. Furthermore, MCAs took the continuous process of advocacy during villagers' social events.

In each village, a village health committee was established from the already available and acceptable community leaders. The aim was to support, facilitate and supervise the work of their MCA.

### Assessment method

Three tools were used to collect data to evaluate the outcome both before (on May 2007) and after (on January 2008) intervention; pre-coded and pre-tested questionnaire (for household survey (HHS)), focus group discussions guide (for focus group discussions (FGD) with community leaders), and structured interview (SI) guide (for interview with MCAs). The HHS targeted to evaluate the treatment seeking behaviour, malaria parasitaemia prevalence, and use of preventive measures; whereas FGD was concerned with community acceptability of the HMM and MCA. On the other hand, the SI was conducted to assess MCAs acceptability, views and barriers regarding the intervention. Records and reports analysis -including supervisory reports- were used to evaluate the adherence to the study protocol and assessing the HMM system for supplies, distribution and storage. Using EPIInfo software, 10 villages were randomly selected for the survey and FGDs. In each village 20 households were randomly chosen where all their inhabitants were then targeted by the survey questionnaire. FGDs with community leaders were conducted in the same survey villages.

### Data management

Trained social workers were used to collect data. Data was compiled and entered using SPSS version 11.5 and excel software packages. Appropriate tests (*x*^2 ^and *t *tests) were used to compare between pre- and post-intervention results.

### Ethical considerations

The study received ethical clearance of the Federal Ministry of Health, Sudan. Informed consent was taken from both community leaders and individuals. Data were used in high confidentiality and no names were used.

## Results

### The study community

There were 23,733 inhabitants in the selected 20 villages for the intervention. Of them 33.1% were under five and the mean number of household members was 4.4. The female represented 59%. Most of the population surveyed were either children (under the age of 13 years) (40.4%), housewives (17.2%), students (17.2%), or farmers (21.5%); and 70.2% of them were non-educated while only 2.6% and 0.3% were graduated from the secondary schools and/or university as their highest level of education respectively. Malaria parasitological prevalence (pre-rainy season survey) was found to be 0.1% with *P. falciparum *as the only prevailing species. The proportion of households who had any case of malaria (presumptive diagnosis) during the last season was found to be between 40 – 100% and the number of malaria deaths per village during the last season range between 0 – 4, most of them were children as stated by the community leaders during the FGDs. The majority of the population sought treatment for the last episode of fever from public health facilities (98.5%), which were 2 – 30 km far from their home but 66% of it were >5 km long as picked up from the HHS. The means for transportation for referral of cases during rainy season varied between: on foot, donkeys, carts, bicycles, and cars; but some villages were inaccessible at all as the community leaders said. The problems associated with malaria treatment were described by the community leaders as: accessibility to health services, drugs were not cheap and most of the time was difficult to find, treatment was on clinical bases, and treatment sometimes was not satisfactory and patients needed to be referred elsewhere.

Mosquito nets were available in more than one third of surveyed households, of them more than a halve were insecticide-treated (ITNs) and the majority were long-lasting (LLINs). Conversely, the usage did not match the availability (Table 1).

**Table 1:** Background information of the surveyed population, Um Adarah area, 2007

**Variables**	**Pre-intervention** N = 892
Age in years – Mean (SD, min – max)	17.8 (17.7, 0.01–95)

Family size – Mean (SD, min – max)	4.4 (1.7, 1–10)

Female ratio	59.0%

Under fives ratio	33.1%

**Occupation:**	
Housewives	17.2%
Child (*under the age of 13 years*)	40.4%
Student	17.2%
Farmer	21.5%
Officer	1.0%
Others	2.8%

**Level of education:**	
Non-educated	70.2%
Religious school	4.7%
Basic school	22.2%
Secondary school	2.6%
University	0.3%

Parasitic **prevalence**	0.1%

**Place of treatment:**	
Public	98.5%
Private	1.0%
At home	0.5%

**Distance to health services:**	
<5 km	34.0%
5–10	22.5%
>10	43.5%

**Use of preventive measures**	
Windows screened (N = 200)	2.0%
Mosquito nets available (N = 872)	34.8%
The available nets are ITNs (N = 436)	59.0%%
ITNs available are LLINs (N = 318)	95.1%%
Usage of net last night (N = 427)	2.9%

### MCAs performance

MCAs were proposed and selected by village committees. If there was any person with previous health experience then no need for new person to work as MCA. The information gained from SIs with the 20 selected MCAs determined that all of them were male and their mean age was 38.2 years. The educational level varies between: religious school (5%), primary (35%), intermediate (25%), and secondary (35%); where the main profession was community health worker (40%), farmer (50%), merchant (5%), and employee (5%). Forty five percent of them have previous experience of treating malaria (some as community health workers and others gained knowledge by self trials and experience). All health workers of the MCAs had other profession to do; either farmer, merchant or live stock herder.

With regards to adherence to project guidelines as evaluated by supervisory visits and records and reports analysis, only one MCA was not adhered to the treatment guidelines (hence excluded from analysis where appropriate), but all MCAs' approach in managing patients and referring severe cases were accepted (Table 2). Two MCAs had a problem in stock management and usage and interpretation of RDTs, which did not significantly vary in terms of having previous experience in malaria treatment or level of education (P values 1.00 and 0.66 respectively). Six MCAs (30%) did not rely on RDTs results and treating patients with negative RDT results on their clinical sense equating fever with malaria, which did not significantly vary in terms of having previous experience in malaria treatment or level of education as well (P value 0.64 and 0.49 respectively).

**Table 2:** MCAs adherence to project guides, Um Adarah area, 2007

**Skills/criteria**	**Frequency** **N = 20**	**%**
MCAs complying with the treatment guidelines	19	95%

MCAs managed uncomplicated malaria as per guideline	20	100%

MCAs complying with referral instruction (severe malaria and others)	20	100%

RDTs usage and interpretation by MCAs was accepted as per supervisory visits	18	90%

MCAs rely on RDTs in treating patients	14	70%

MCAs ability to manage the stock of drugs and RDTs was accepted	18	90%

Registration and reporting was accepted	20	100%

MCAs Place of work (clinic) was accepted	19	95%

MCAs attending all scheduled meetings	17	85%

All MCAs registration and reports were accepted. Most (95%) of the MCAs adhered to the project design in attending scheduled meetings and submitting their monthly reports. That was 85% (17/19) of them was attending the meetings on regular bases, and 10% (2/20) had accepted reasons for their absence, delay in meetings, or late reports submission.

Moreover, MCAs (65% of them (13/20)) had initiated educational and health promotional activities and campaigns which were appreciated and supported by their communities.

From those who had previous experience in malaria treatment, 37.5% (3/8) had problem in convincing clients that they had malaria before implementation of the project, compared to 5.3% (1/19) who had the same problem after the implementation (P. value 0.064). On the other hand, 50% (4/8) faced a problem of persuading someone that he had no malaria; compared to 21% (4/19) for post intervention group that used RDTs (P. value 0.02) as picked up through SIs. Eighteen out of nineteen (94.7%) of the MCAs did accept seeing patients at night for consultation and this did not harm him in any way. A similar proportion (94.7%) of MCAs felt the social and spiritual outcome of this work was persuasive for and appreciated by them and their families. Vice versa, 36.8% (7/19) was assuming that the financial outcome (the consultation fees) of this work was not satisfied for them, and all of them felt that the financial gain was not enough to the degree that lead them to leave their original work.

Village health committees (95%, 19/20 of committees) together with their villagers supported their MCA by either providing or building a room to be a clinic. As per the SIs findings, most (73.7%, 14/19) of village health committees were active in visiting their MCA in his clinic and/or house and discussing with him his work and his problems; since 68.4% had visited their MCA at least once every two months and only 21.1% had never visited their MCA. This was reflected by the perception of the 84.2% (16/19) of the MCAs that their village health committees had a positive impact in his work (Table 3).

**Table 3:** MCAs post intervention perception and attitude, Um Adarah area, 2007

**Skills/criteria**	**Frequency** **N = 20**	**%**
MCAs carried out awareness activities at community level	13	65%

MCAs experienced any problem to convince someone he had malaria	1	5%

MCAs experienced any problem to convince someone he had no malaria	4	20%

MCAs accepted patients at night	18	90%

MCAs agreed that doing this work is persuasive to him and his family	18	90%

MCAs agreed that the financial outcome did satisfy him and his family	7	35%

Village committees visited the MCAs	16	80%

Village committee positively added to MCAs	16	80%

The system for ACTs and RDTs supply and distribution were found to be effective since no MCA reported stock out or complaint of difficulties in replenish their stock or even having difficulties in access to that.

### Treatment-seeking behaviour

The prevalence of households with fever case within the last two weeks prior to the survey was identified as 24% and 8.5% of the total households surveyed (200 households) in the pre- and post-intervention surveys respectively (P. value 0.000). Out of those houses with fever cases, 83.3% (40/48) of the mothers sought treatment for their febrile child prior to the implementation of the HMM project which was increased to 100% (17/17) after the implementation (P. value 0.099). Of them, 25% (10/40) sought treatment within their villages, compared to 64.7% (11/17) for pre- and post-project implementation (P. value 0.004). Concerning severe malaria, two and one cases were noticed within the last six months before and after the availability of HMM services respectively. The number of deaths in the last season before the project was 61, and it was only one after implementation of the project (P. value 0.000). Of them, 100% and 0.0% were <5 years old (P. value 0.016) for pre and post intervention groups respectively.

### Patients treated with MCAs

The HMM project served a population of more than 23,000 residents. A total of 3,745 patients were treated during the period June – December 2007. Of them 17.6% (659 patients) were children under 5 years. MCAs during the same period referred 35 (0.9%) cases to the nearest hospital, diagnosing them as having other febrile conditions rather than malaria. MCAs reported 7 (0.2%) deaths in their respective villages with variable causes of deaths. MCAs also reported 7 (0.2%) cases as having mild side effects coincided with ACT use.

### Satisfaction with the project

When the issue of HMM was discussed with the village community leaders in an organized focus groups, all villagers said that they knew that there is an MCA available in their village to treat malaria cases and give advice for other conditions. They got the message through different channels like the village health committees, social gatherings, mosques, meeting with other villagers, MCAs themselves, neighbours and relatives within the village, patients those were treated by MCA, and the graduation celebration of the MCAs. The message that they receive was that the diagnosis and treatment of malaria was within the villages from a trained personnel. Those who made use of the HMM services acknowledged it because it was easy, nearby and cheep service. As well, they mentioned that the selected MCAs were accepted by them because of their honest, the way they were dealing with their patients, activity, had a time for their patients and their own work, from the villagers, and knew what to do. No one described any obstacles hindering the continuation of the project; but they ask for expansion of the project to involve other diseases. All villagers found to be eager for the continuation of the project after the end of the pilot due to the burden of disease that was released from their communities.

## Discussion

HMM was proud to be an effective method in reducing disease burden and solving the problem of access to health services [21-26]. Hence, the scope of this study beyond this goal was to assess the feasibilty and acceptbility of this method in Sudan's situation. Moreover this study was built on ACTs and RDTs use by volunteers.

Admitedly, the only suitable interevention regarding malaria control in rural poor setings and far to reach communities are early and proper case management and high coverage and usage of ITNs. This study revealed that the coverage of ITNs is somewhat low but allmost all of them were LLINs. Beside that, the usage was very limited which is an open area for promotional interventions.

Having previous experience in managing malaria or being educated did not significantly tend to affect the outcome of MCAs performance or the rely on RDTs result in treating fever cases, despite the general variation in taking the RDTs results in mind whent diagnosing and treating fever. Chandler found that patient demand was not found to be driving the over-prescription of anti-malarials. To the contrary, the involvement of patients may provide an opportunity to improve prescribing practice if their expectations for test and treatment in line with test results can be effectively communicated [27]. Nevertheless, RDTs were found to have less significant effect in conveincing febrile patients with their diagnosis. With its simple use and despite the incremental cost compared with microscopy, RDTs proved its ability in increasing the percentage of patients correctly treataed; but cost-effectiveness would be worse if prescribers do not comply with test results [28].

MCAs proof their ability to record and report cases and in using the format for registration and reporting. On the other hand, the project increased the treatment seeking behavior and increased the access to anti-malarial drugs at village level. Such results were identified elsewhere in similar settings [5,7,22,26,29].

Despite the low income of the MCAs from the HMM project, MCAs and their families were found to be saticfied with the social and spiritual outcome of providing such a services for their comminties. The fact that MCAs did not leave their original proffesions, can be an important factor for the sustainability of the project and should be followed in the future design for such initiatives.

The role of the community in sellecting their MCAs was found to be importnt. As well, the invovement of villagers in supervising and supporting the project was of graet value in keeping the project implemeted in ease. Furthermore, the community were satisfied by the projet implementation and had the interest of expansion of the project to cover other common diseases. Community satisfaction did not varies with MCAs who had previous experience in managing malaria or with the others.

With regards to MCAs commitment with the HMM programme plan, they were found to be very adherent to meeting attendance and reporting dates. When coming to ACTs and RDTs supply, storage and distribution system; it was proved to be satisfiing. Furthermore, MCAs has performed well in intiation of health promotional and community mobilization activities in their communities. One of the good ways to advocate for the project and mobilize the community to uptake the HMM services were found to be the interpersonal communication methodes and tools done by the investigators and the MCAs themselves.

## Conclusion

Making use of this method in implementing the HMM project in Sudan seems to be promising. The use of the RDTs in such condition is fundamental since it help in supporting the diagnosis and the trust in volunteers. Although the project needs some adaptation, but its general feasibility and acceptability was proved. The expansion of the project to include Integrated Management of Childhood Illnesses (IMCI) targeted diseases and in particularly those causing fever looks sound in reducing the overall morbidity and mortality.

## Competing interests

The authors declare that they have no competing interests.

## Authors' contributions

KAE and EMM have designed the methodology, done the analysis and wrote the manuscript. KAE trained the working teams, and designed the study materials. TA and AHE were involved in the study design, implementation and analysis. MAM and AHE were the focal persons for the training, monitoring and quality assessment of the RDTs and the study drugs respectively. SHA was the focal person for the surveys, interviews, FGDs and social mobilization in the study. IA has contributed in the study design, monitoring and editing of the manuscript. All authors read and approved the manuscript.
